# Mapping of mothers' suffering and child mortality in Sub-Saharan Africa

**DOI:** 10.1038/s41598-021-98671-9

**Published:** 2021-10-01

**Authors:** Bayuh Asmamaw Hailu, Gebremariam Ketema, Joseph Beyene

**Affiliations:** 1 Monitoring and Evaluation, Wollo University, Dessie, Ethiopia; 2 Department of Pharmacy, Collage of Medicine and Health Science, Wollo University, Dessie, Ethiopia; 3 Department of Health Research Methods, Evidence, and Impact, McMaster University, Hamilton, Canada

**Keywords:** Health care, Medical research, Risk factors

## Abstract

Child death and mothers who suffer from child death are a public health concern in Sub-Saharan Africa. The location and associated factors of child death and mothers who suffer child death were not identified. To monitor and prioritize effective interventions, it is important to identify hotspots areas and associated factors. Data from nationally representative demographic and health survey and Multiple Indicator Cluster administrated in 42 Sub-Sahara Africa countries, which comprised a total of 398,574 mothers with 1,521,312 children. Spatial heterogeneity conducted hotspot regions identified. A mixed-effect regression model was run, and the adjusted ratio with corresponding 95% confidence intervals was estimated. The prevalence of mothers who suffer child death 27% and 45–49 year of age mother 48%. In Niger, 47% of mothers were suffering child death. Women being without HIV knowledge, stunted, wasted, uneducated, not household head, poor, from rural, and from subtropical significantly increased the odds of the case (P < 0.05). The spatial analysis can support the design and prioritization of interventions. Multispectral interventions for mothers who suffer from child death are urgently needed, improve maternal health and it will reduce the future risk of cases.

## Introduction

The birth of a child is a time of wonder and celebration within the mother because children are a valuable resource in developing countries. Due to the difficulties in the life of low-income country^1^, the only goal for most families is to raise children. Mothers ignore their own hunger in order to feed their children^2^. Children who have the opportunity to learn serve their families in their spare time, else they serve full-time. When they grow up, they do different jobs and help as a source of income for the family. When mothers lose the support of their children in their retirement, may be they would survive independently, but they will be starve and forced to wander in streets. As a result, for mothers, the death of older children is more likely to be intolerable than that of infants. Even if the community does not isolate, the mothers will isolate themselves. When a child dies, the mothers’ dream and hope die too^3^ and have heavy bereavement. Due to more bereavement of the mother, there is a high risk of grief. Grief due to child death in the family may be more severe, varied, and long-term^4^.

In Sub-Sahara Africa (SSA), the death of a child affects all mothers in the same way, most of them dealing with grief, because it influences by the culture, personality, lifestyle, family relationship, and their ultimate goal. According to UNICEF’s September 2020 report in the past three decades, the world has made remarkable progress in child survival, and millions of children have better survival chances than in 1990 (1 in 11), compared to 2019 (1 in 27) children died before reaching age five. In addition, progress in reducing child mortality rates has been accelerated over the years 2000–2019 compared to the 1990s. Approximately 14,000 children per day or 5.2 million children under the age of five died in 2019^5^. More than half of these deaths have occurred in sub-Saharan Africa^5^.

Continuing efforts to reduce not only under-five and infant mortality but also adolescent mortality will reduce the risk of child deaths and mothers suffering from child death and the consequences of grief of a mother. In this shadow of the global health community, usually focuses on mothers with under-five child mortality, the rest are all those grieving parents who have never received any attention. The location and associated factors of child death and mothers who suffer child death were not identified. This study is important for the development of programs in SSA, to support the high risk of bereaved mothers and grieving parents as they navigate life after the loss of a child and also shows the place of unfairly concentrated loss of children, hotspot areas and characteristics of this high-risk group in SSA.

## Results

### Spatial epidemiology

**Figure 1:**
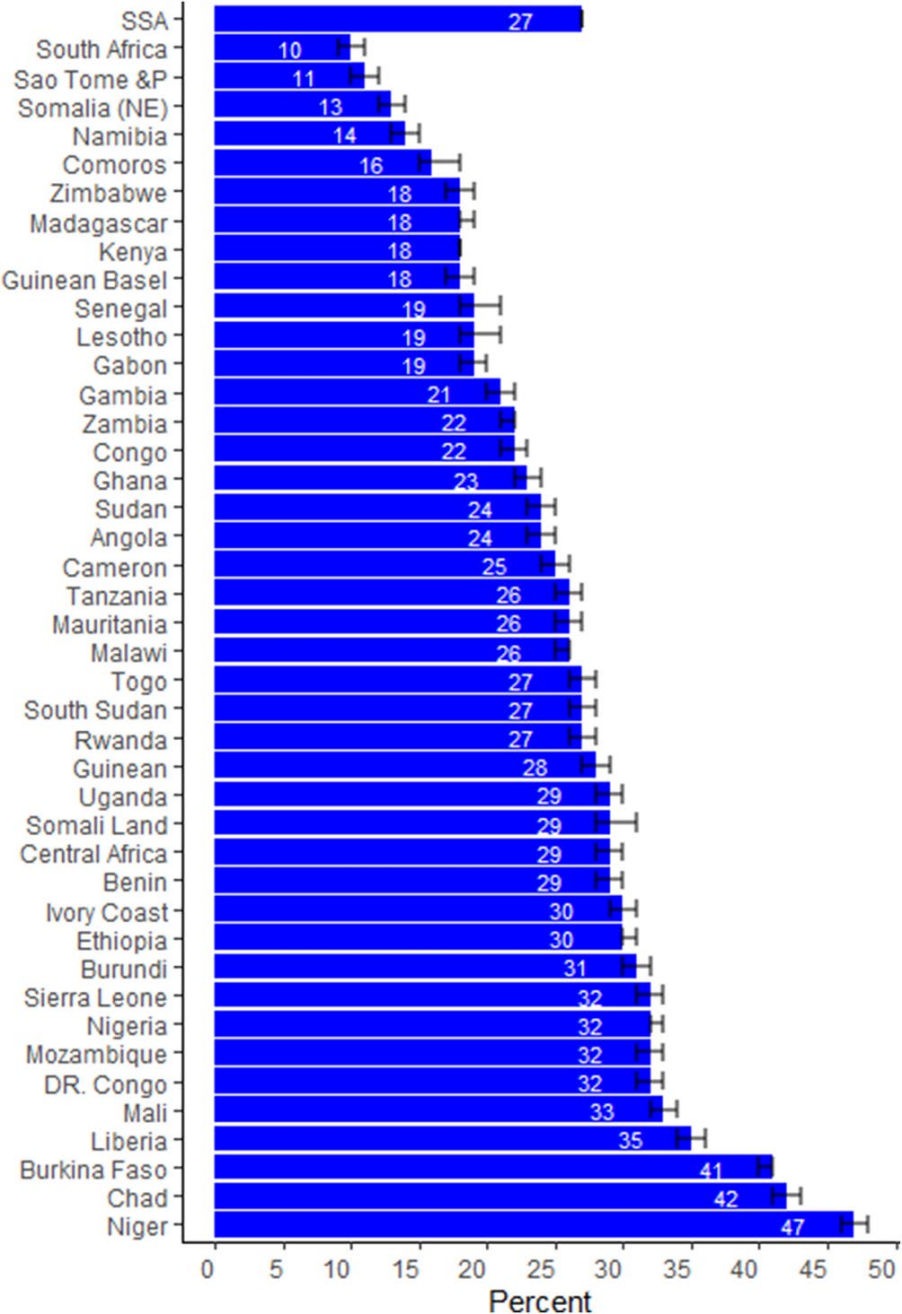
The proportion of women who suffer at least one child death with the confidence interval.

A total of 398,574 mothers under the age of 50 and 1,521,312 children born of these women were analysed in 42 countries, of which 179,589 (12%) children’s died from 108,334 (27%) of mothers. On average a woman had 3.8 children. From child death experienced mothers on average each mother lost 1.7 children. The highest prevalence of mothers who lost at least one child was in Niger (47%), Chad (42%), and Burkina Faso (41%). The lowest proportion of women who suffer in child death were South Africa and Sao Tome and prince women, which account out of 10 women about one women were suffer in child death (Fig. 1; Table 1).

**Figure 2:**
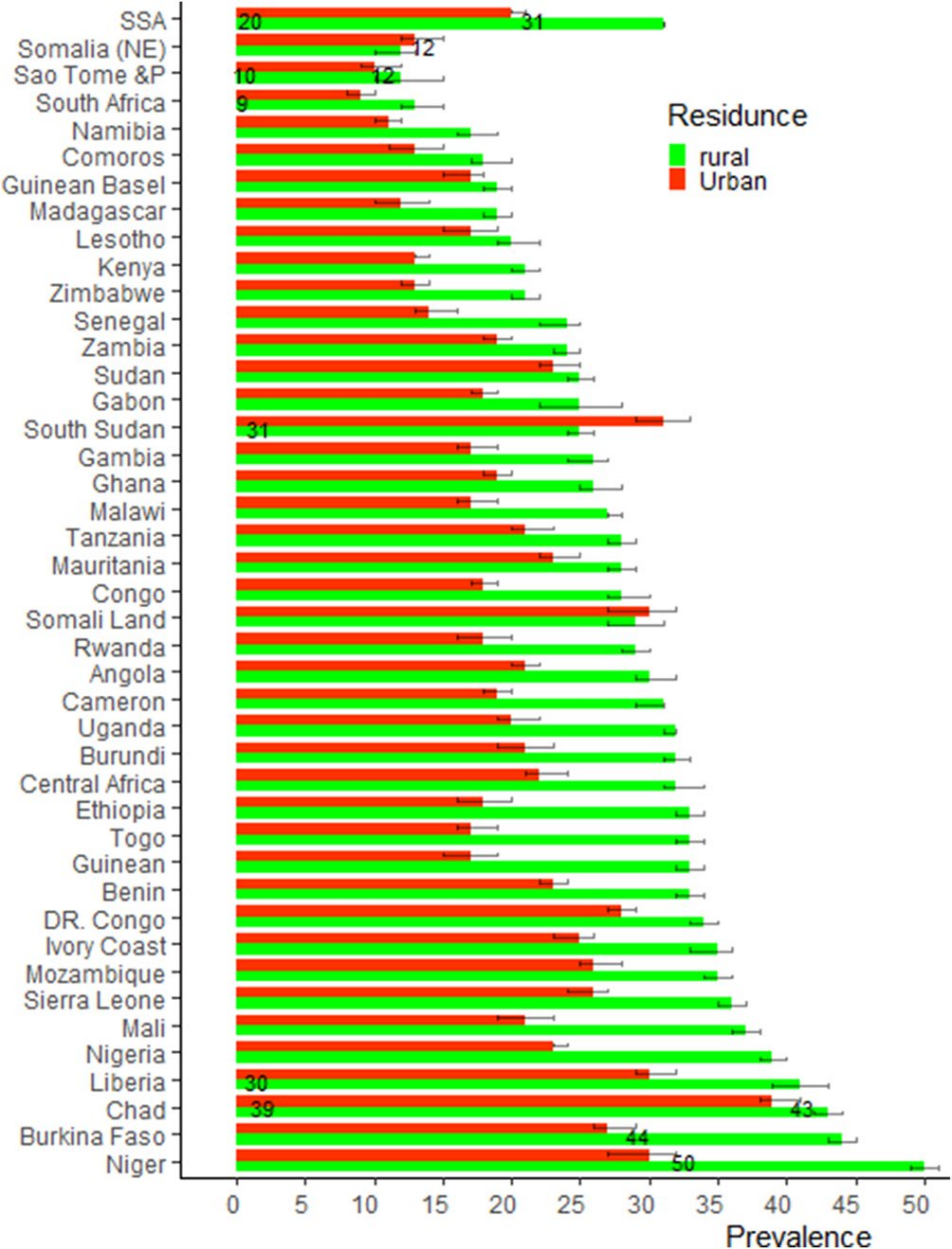
The proportion of women who suffer at least one child death by residence: the red and the green color indicate the proportion of urban and rural, respectively mothers who suffer in at least one child with confidence interval.

Almost all countries women in rural areas were higher proportion of contributing for at least one child death compared to urban area except both Somalia and South Sudan. In average 31% of women from rural area and 20% women from urban area were contribute for at least one child death. The highest proportions of mothers from rural area who suffer in at least one child death were in Niger which account half of them were suffer. The next highest were mother from chad and Burkina Faso which accounts two out of five mother were contribute for at least one child death. Out of five mothers in urban areas in Chad two of them (39%) were suffer in at least one child death, the next highest proportion women urban areas in South Sudan (31%) and Liberia (30%) were suffer in child death. The lowest contribution of mother in child death were Sao Tome and prince, South Africa, and Somalia women which accounts out of 10 women from those country about one of them were suffer in at least one child death (Fig. 2; Table 1).

The three countries with the highest prevalence of child death were Niger (20%), Burkina Faso (17%), and Liberia (17%). Child death experienced mothers from Niger, Chad, Nigeria, Angola, South Sudan, Liberia, Burkina Faso and Somali Land; each mother lost an average of two children (Table 1).

### Among women age 45–49 years

**Figure 3:**
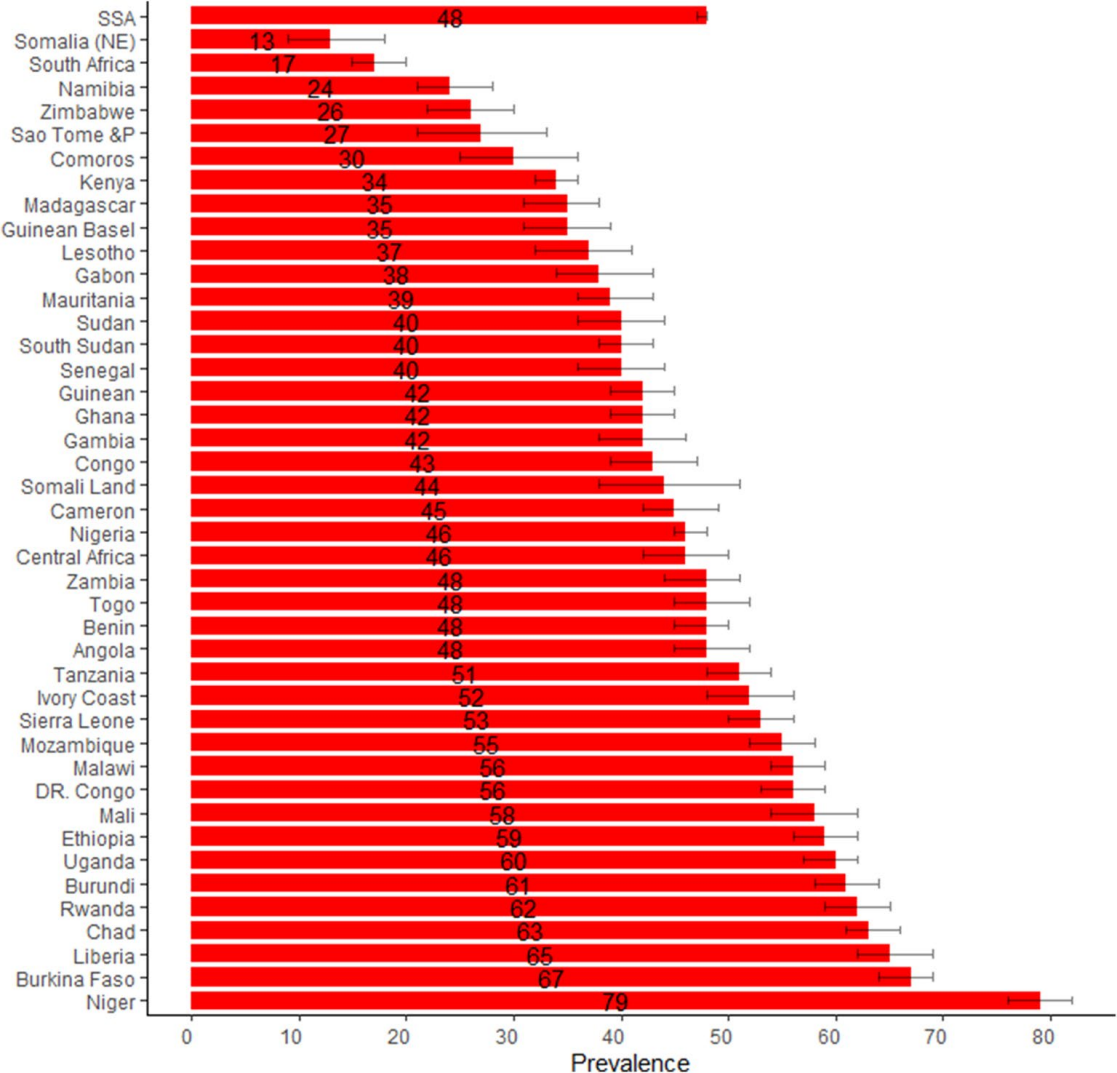
The proportion of women aged of 45–49 years who suffer at least one child death with confidence interval. All analysis of bar graph and confidence interval was used R soft wear (version R 4.1.0).

 The average birth rate was 6 children per one mother and the highest birth rate countries are Niger and chad (each women in average birth 8 children), the lowest was South Africa which account 3 children in each mother. About half of (48%) mothers whose age 45–49 suffered child death and from 15 countries above half of women suffered child deaths. Out of five women in Niger four of them were suffer in child death, which is the highest proportion compared to other countries. The next highest proportion of mother who suffer in at least one child death in this age group were Burkina Faso (67%), Liberia (65%), Chad (63%), and Rwanda (62%) (Fig. 3; Table 1).

**Figure 4:**
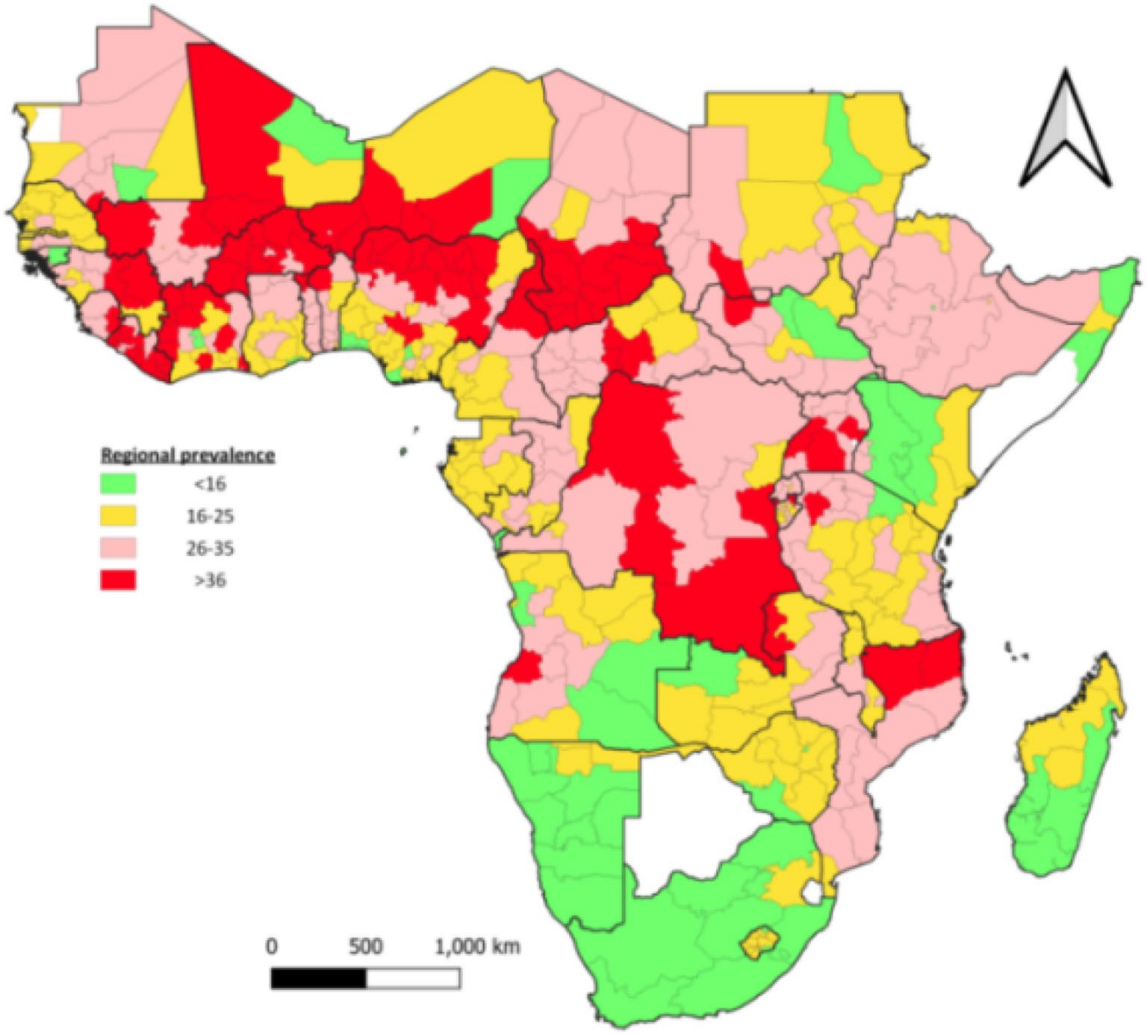
Subnational prevalence of mothers who lost at least one child due to death: the color from green to red shows an increasing prevalence of women who have lost at least one child. This analysis was carried out by QGIS 3.16.

SSA mothers who suffer from child death are a serious public health concern. Differences of up to 20 percentage points in prevalence within the same country were common. Mother who had lost their child due to death is high in most Western and some Central SSA regions (Fig. 4).

#### Hot/cold spot analysis

**Figure 5:**
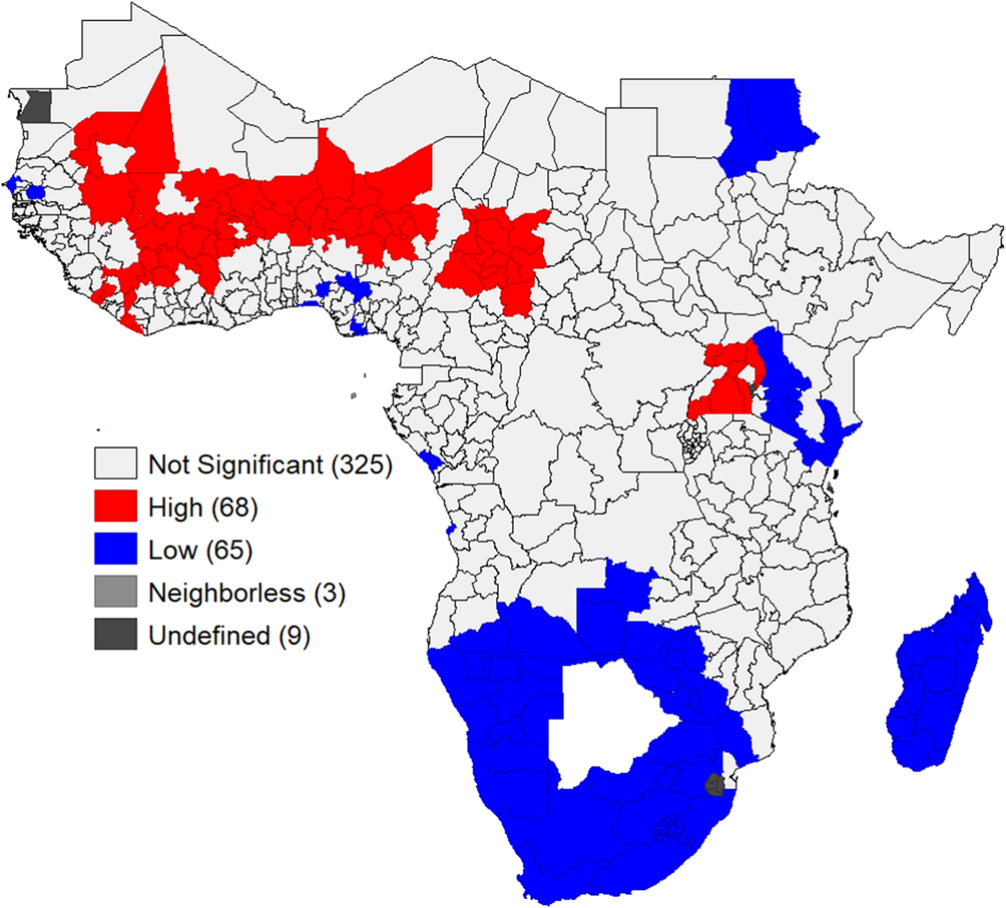
Hot-spots and cold-spots of women who suffer child death: each polygon on the map represents a single zone area with mothers who suffer child death. High (red colour) means high (hot spot) of mothers who suffer from child death. Low (blue colour) shows a low (cold spot) of mothers who lost at least one child. To perform this analysis was used GeoDa GIS version 1.14.

Hot spot analysis is performed by means of a statistical test. The red color (hot spot) shows a higher risk of mothers suffering from at least one child death. Out of the total regions in SSA, 68 of them of were the hot spot regions compared to its neighbour. Most of those regions were found in Western SSA. In the cold spot 65 regions were identified, Most of them obtained in Southern SSA (Fig. 5).

#### High-value cluster using the Poisson model

**Figure 6:**
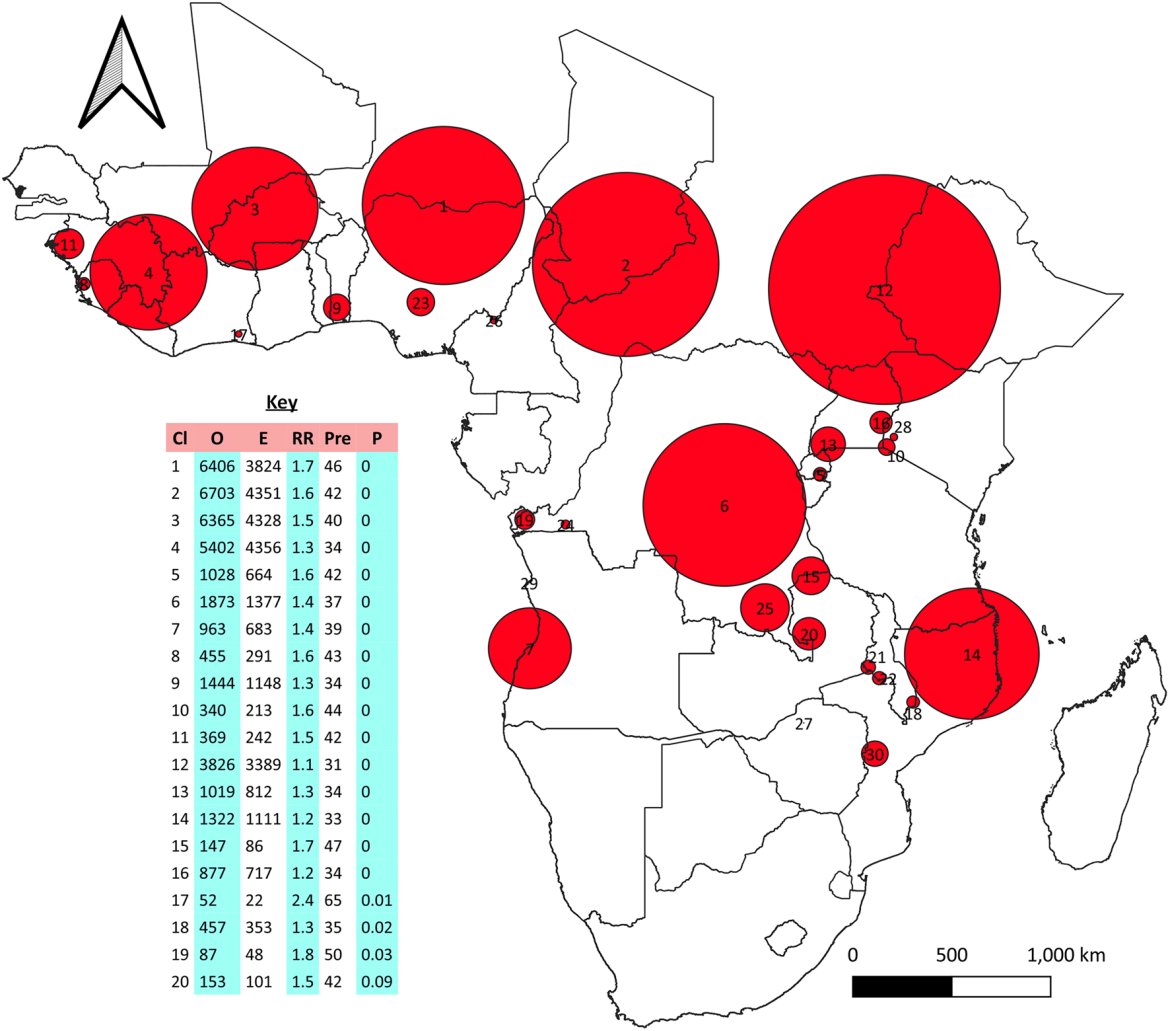
High-value clusters: the red circular shape shows the windows of the hotspots of mothers who have experienced at least one child's death. *Cl* cluster number on the map, *O* observed cases in the clusters, *E* expected cases in the cluster, *RR* relative risk, *Pre* prevalence, *P* P value. To conduct this analysis was used SaTScan v9.6 and QGIS.

Nineteen statistically significant clusters have been identified using SaTScan (P < 0.05). The primary circular window (cluster) of mothers who suffer from child death was found in Nigeria. In this cluster, 3824 cases expected but found 6406, 46% of mothers were suffered from child death with a Relative Risk (RR) of 1.7.The second major cluster was located in Chad. The expected number of cases in this cluster was 4351, but 6703 cases were identified. The relative risk was 1.6, with 42% of mothers suffering from child death. The third most likely significant clusters were found in Burkina Faso and Mali. In this cluster, 4328 cases were expected, but 6365 cases were identified, with RR 1.5 and 40% of mothers suffering from child death (Fig. 6).

#### Spatial interpolation

**Figure 7:**
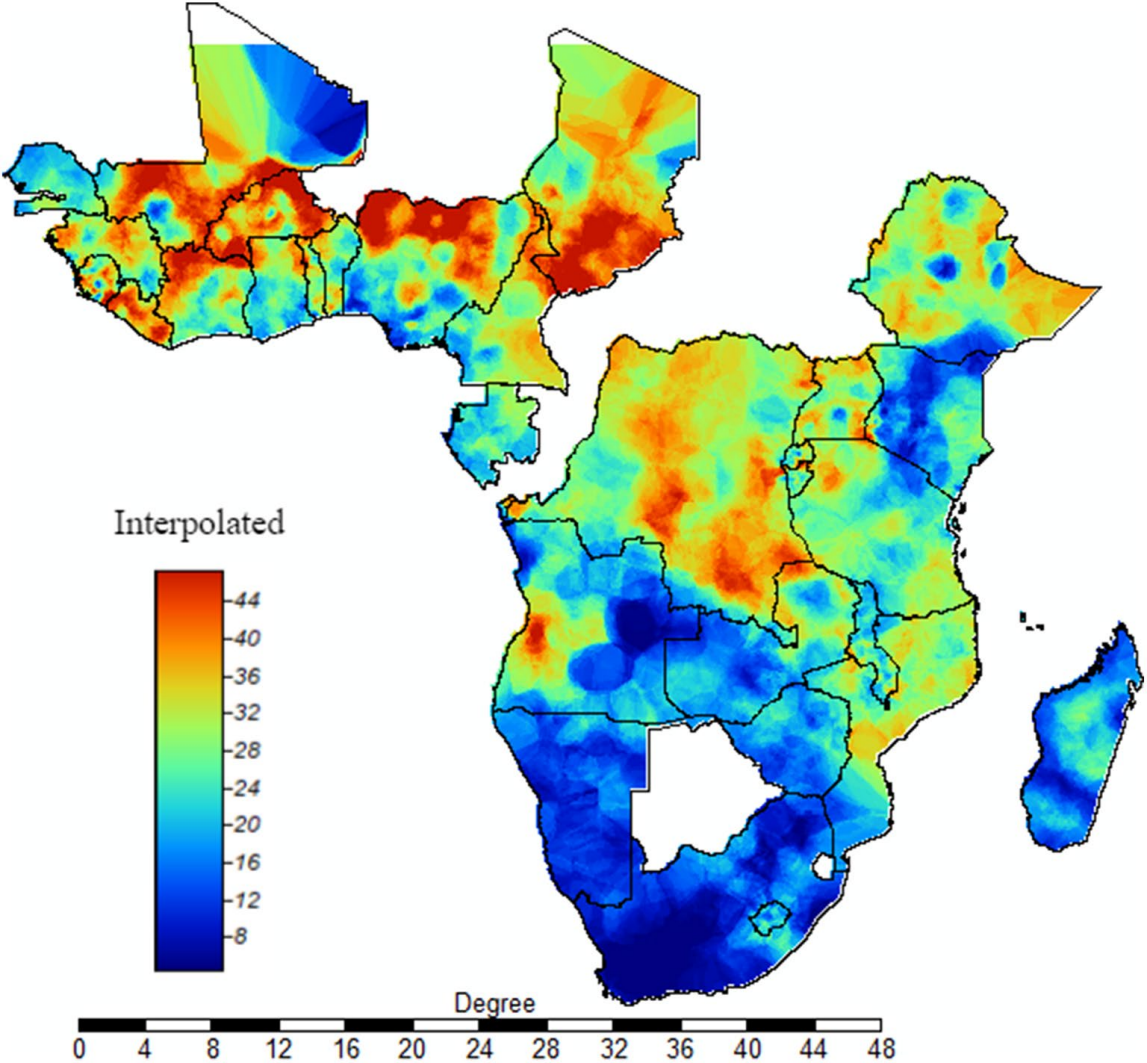
Interpolation of mothers who suffer at least one child death: the interpolated continuous images provided by the interpolation ordinary kriging. The colour through bold blue to bold red indicates an increase in the prevalence of mothers who suffer from child death. This analysis was carried out at SAGA GIS.

Our spatial interpolation revealed that mothers who have suffer from child deaths have been a serious public health problem in most areas of SSA, with the exception of southern SSA countries. Most areas of Western SSA, Democratic Republic of Congo, Ethiopia, Uganda, and Mozambique and some areas of Angola and Tanzania had extremely high burden of mothers who lost at least one child (Fig. 7).

#### Model comparison

Without any covariant, the ICC value for child death and mothers who suffer from child death were 8.6% and 10.5%, respectively. This illustrated that the multilevel analysis was more appropriate for both cases because ICC is > 5%. The Poisson regression variance (0.843) was greater than the mean (0.447), indicating that the data were over dispersed. Thus, Poisson regression with over dispersion is treated as a negative binomial regression. According to Log's likely hood results, the best fitness was the negative binomial regression (Table 2).

**Table 1:** Spatial distribution of sample size case prevalence, birth rate, ratio, residence and older age in child death and mother suffering of child death.

Country	Data	Number of sample (%of case)		BR	Ratio	%MD (95% CI)		45–49 year	
	Source, year	MT	CT		CD/MD	Rural	Urban	%MD (95% CI)	BR
SSA		**398,574 (27)**	**1,521,312 (12)**	**3.8**	**1.7**	**31 (31,31)**	**20 (20,21)**	**48 (47,48)**	**6**
Niger	DHS, 2012	9460 (47.1)	46,719 (20.1)	4.9	2.1	50 (49,51)	30 (27,32)	79 (76,82)	8
Chad	DHS, 2014/5	14,143 (42.1)	69,245 (16.3)	4.9	1.9	43 (42,44)	39 (38,41)	63 (61,66)	8
Burkina Faso	DHS, 2010	13,270 (40.7)	56,330 (17.0)	4.2	1.8	44 (43,45)	27 (26,29)	67 (64,69)	7
Liberia	DHS, 2013	7186 (34.7)	26,665 (16.9)	3.7	1.8	41 (39,43)	30 (29,32)	65 (62,69)	7
Mali	DHS, 2018	8362 (33.4)	35,429 (13.7)	4.2	1.7	37 (36,38)	21 (19,23)	58 (54,62)	7
Nigeria	DHS, 2018	29,949 (32.4)	126,538 (14.6)	4.2	1.9	39 (38,40)	23 (23,24)	46 (45,48)	7
Mozambique	DHS, 2011	10,761 (32.2)	39,896 (15.2)	3.7	1.7	35 (34,36)	26 (25,28)	55 (52,58)	6
DR. Congo	DHS, 2013/4	13,894 (32.1)	57,487 (13.4)	4.1	1.7	34 (33,35)	28 (27,29)	56 (53,59)	7
Sierra Leone	DHS, 2019	11,457 (31.7)	39,764 (15.5)	3.5	1.7	36 (35,37)	26 (24,27)	53 (50,56)	6
Burundi	DHS, 2016/7	11,359 (31.0)	46,871 (12.1)	4.1	1.6	32 (31,33)	21 (19,23)	61 (58,64)	7
Ethiopia	DHS, 2016	10,587 (30.5)	44,596 (11.7)	4.2	1.6	33 (32,34)	18 (16,20)	59 (56,62)	7
Ivory Coast	DHS, 2011/12	7371 (30.1)	26,982 (13.4)	3.7	1.6	35 (33,36)	25 (23,26)	52 (48,56)	6
Somali Land	MICS, 2011	3316 (29.1)	16,811 (10.5)	5.1	1.8	29 (27,31)	30 (27,32)	44 (38,51)	7
Benin	DHS, 2017/8	11,783 (28.9)	45,856 (11.7)	3.9	1.6	33 (32,34)	23 (22,24)	48 (45,50)	6
Central Africa	MICS, 2010	7197 (28.8)	27,430 (11.4)	3.8	1.5	32 (31,34)	22 (21,24)	46 (42,50)	6
Uganda	DHS, 2016	13,693 (28.8)	57,029 (10.7)	4.2	1.5	32 (31,32)	20 (19,22)	60 (57,62)	7
Guinean	DHS, 2018	7689 (28.0)	28,189 (12.2)	3.7	1.6	33 (32,34)	17 (15,19)	42 (39,45)	5
Rwanda	DHS, 2014/5	8828 (27.0)	30,725 (12.4)	3.5	1.6	29 (28,30)	18 (16,20)	62 (59,65)	6
South Sudan	MICS, 2010	7345 (26.5)	29,085 (12.3)	4	1.8	25 (24,26)	31 (29,33)	40 (38,43)	6
Togo	DHS, 2013/4	6845 (26.6)	24,664 (11.4)	3.6	1.5	33 (32,34)	17 (16,19)	48 (45,52)	6
Mauritania	MICS, 2015	8119 (26.0)	33,033 (9.7)	4.1	1.5	28 (27,29)	23 (22,25)	39 (36,43)	6
Tanzania	DHS, 2015/6	9893 (25.7)	36,347 (10.4)	3.7	1.5	28 (27,29)	21 (20,23)	51 (48,54)	6
Malawi	DHS, 2011	19,030 (25.6)	68,484 (11.0)	3.6	1.5	27 (27,28)	17 (16,19)	56 (54,59)	6
Cameron	DHS, 2012	9482 (24.6)	34,985 (10.6)	3.7	1.6	31 (29,31)	19 (18,20)	45 (42,49)	6
Sudan	MICS, 2014	11,528 (24.2)	51,066 (8.3)	4.4	1.5	25 (24,26)	23 (22,25)	40 (36,44)	7
Angola	DHS, 2015/6	10,793 (24.2)	41,123 (12.2)	3.8	1.9	30 (29,32)	21 (20,22)	48 (45,52)	6
Ghana	DHS, 2014	6458 (22.6)	22,139 (9.0)	3.4	1.4	26 (25,28)	19 (18,20)	42 (39,45)	5
Congo	DHS, 2011/12	8286 (21.9)	26,841 (9.2)	3.2	1.4	28 (27,30)	18 (17,19)	43 (39,47)	5
Zambia	DHS, 2018/9	10,318 (21.7)	37,627 (9.0)	3.6	1.5	24 (23,25)	19 (18,20)	48 (44,51)	7
Gambia	DHS, 2013	6784 (21.4)	25,896 (8.2)	3.8	1.5	26 (24,27)	17 (16,19)	42 (38,46)	6
Senegal	DHS, 2019	5478 (19.5)	20,106 (7.2)	3.7	1.4	24 (22,25)	14 (13,16)	40 (36,44)	6
Lesotho	DHS, 2014	4542 (19.4)	11,575 (9.7)	2.5	1.3	20 (19,22)	17 (15,19)	37 (32,41)	4
Gabon	DHS, 2012	5968 (18.5)	18,906 (7.6)	3.2	1.3	25 (22,28)	18 (17,19)	38 (34,43)	6
Madagascar	MIS, 2016	8339 (18.4)	27,943 (8.5)	3.4	1.5	19 (18,20)	12 (10,14)	35 (31,38)	6
Zimbabwe	DHS, 2015	7309 (18.3)	21,677 (8.2)	3	1.3	21 (20,22)	13 (12,14)	26 (22,30)	4
Guinean Basel	MICS, 2018/9	7651 (18.1)	25,045 (8.2)	3.3	1.5	19 (18,20)	17 (15,18)	35 (31,39)	5
Kenya	DHS, 2014	22,974 (18.0)	77,125 (7.5)	3.4	1.4	21 (20,22)	13 (13,14)	34 (32,36)	5
Comoros	DHS, 2012	2940 (16.5)	11,562 (6.1)	3.9	1.4	18 (17,20)	13 (11,15)	30 (25,36)	6
Namibia	DHS, 2013	6222 (13.9)	16,971 (6.5)	2.7	1.3	17 (16,19)	11 (10,12)	24 (21,28)	4
Somalia (NE)	MICS, 2011	3588 (12.6)	15,278 (4.7)	4.3	1.6	12 (10,13)	13 (12,15)	13 (9,18)	5
Sao Tome &P	MICS, 2014	2224 (10.8)	7336 (4.2)	3.3	1.3	12 (10,15)	10 (9,12)	27 (21,33)	5
South Africa	DHS, 2016	6153 (10.2)	13,936 (5.6)	2.3	1.2	13 (12,15)	9 (8,10)	17 (15,20)	3

*MD* the number of mothers who are suffering child death, *MT* total number of mothers, *CD* total number of child death, *CT* total number of child birth, *BR* birth rate.

#### Mixed effect logistic regression

After controlling for confounding factors at the individual (maternal) and community levels, HIV knowledge, stunting and wasting, age, education, household size, relationship to household head, wealth, age at first birth, number of unions, residence, source of drinking water, and ecology were statistically significant factors for both child death and mothers who suffer in child death (Table 3).

**Table 2:** Model compression for analysis and checking variability.

	Poisson	Negative binomial	Logistic
Mean	0.447		
Variance	0.843		
Estimate (SE)	0.79	0.83	
Log likely hood	− 74,543	− 72,451	
AIC	149,167	144,984	
P value	< 0.001	< 0.001	
ICC	10.5	10.5	8.6

#### Mothers who suffer from child death

Stunted, wasted, and overlapping (both stunting and wasting in women) mothers who were 21%, 25%, and 43%, respectively, are more likely to suffer by children death than mothers who were neither stunted nor wasted. Repeatedly married mothers are 31% more at risk than once married mothers in child death. Mothers living in rural areas are 17% more likely to suffer child deaths compared to urban mothers. Mothers using an unimproved water source had a 7% higher risk of child death than mothers using an improved water source (Table 3).

#### Child death

The incidence rate for child death from mothers without HIV knowledge were 44% more risk than the incidence rate of the number of child death whose mother have HIV knowledge. The incidence rate of the number of child deaths from mothers aged 20–24, 25–29, 30–34, 35–39, 40–44 and 45–49 were 1.85, 3.8, 5.9, 8.6, 11.6 and 14.5, respectively times higher than the incidence rate of the number of child death from mothers age 15–19 (Table 3).

## Discussion

Child death and mothers who suffer from child death are high in SSA. This finding is in line with other finding^6^. Most parts of western Africa, in some parts of Central and Eastern SSA were high compared to others. Health care services are a key proximate determinant of maternal and child health^7^. In addition, timely and appropriate maternal and child care can provide an opportunity to prevent or manage the causes of child mortality. Maternal health status is directly linked to child mortality^8^. ANC can improve children's and maternal health by identifying, managing, and referring to potential complications. ANC is also related to the prevention, identification and treatment of multiple health problems associated with child mortality^7,9^. The other possible reasons will maternal health, much is known about the consequences of anemia during pregnancy, including the increased risks of low birth weight, preterm birth, and neonatal mortality^10^. The other possible reason for contributing to child death is child anemia^11,12^. It has serious consequences, including child morbidity and mortality^13^. The consequence of malnutrition is high in SSA^14^. In addition, inadequate dietary diversity in children is one of the potential risks of child mortality. A lack of complementary feeding practices is the main cause of under-nutrition, which is a direct cause of child mortality^15,16^.

Maternal HIV knowledge has negative association with child mortality. This finding is in line with other findings^17–19^. The possible reason for this strong correlation is that increased maternal knowledge reduces child death in HIV by protecting mother-to-child transmission during pregnancy, childbirth and post-birth^20^. Child mortality in educated mothers is lower than in uneducated mothers. This finding is similar to that of the other findings^21–24^. Therefore improving maternal education will improve the health of their children and the community^25,26^. A mother's age at first birth is negatively correlated with child death, with a decrease in mother's age at first birth increases the risk of child mortality. Which is similar to the previous studies^22^. Rural children are more likely to die than their counterparts. Potential causes of rural child deaths include access to health centre, reproductive health education, sanitation, quality of drinking water, and so on^27^. Children in relatively poorer households were more likely to died^28^. This is due to inadequate household income, inadequate sanitation, malnutrition, poor access to health care among families. Women who have been repeatedly married higher exposure to the risk of child death. This finding is in line with other findings^29^. This is due to women who have been repeatedly married may have had more children. The number of child increase, the child death increase too^29^.

Finally we recommended that policymakers and other concerned bodies who are working on this serious public health issues in SSA countries should work in collaboration and understanding in order to mitigate the underline problems. Though the child death and mothers who suffer in child death are serious public health issues in each SSA country, priority has to be given in most areas of Western, some areas of Central and Eastern SSA countries.

As the problems of child death and mothers who suffer from child death are relatively serious in women who do not know about HIV, stunted, wasted, not house wife, repeatedly married and older age; and in households of large family size, unimproved water and sanitation; from communities who indwells in rural and low land areas, effective intervention measure should be designed and followed.

**Table 3:** Multilevel regression associated characteristics with child death and mothers suffering child death.

Characteristics	Crude	AOR^a^ (95% CI)	AIRR^b^ (95% CI)
**Lower level (maternal) characteristics**			
HIV knowledge (Ref. Have not knowledge)	1	1	1
Have knowledge	0.49 (0.48, 0.5)	0.52 (0.5, 0.53)**	0.56 (0.55, 0.56)**
Stunting with wasting (Ref. Free)	1	1	1
Only stunting	1.18 (1.15, 1.22)	1.21 (1.16, 1.27)**	1.16 (1.13, 1.2)**
Only wasting	1.84 (1.76, 1.92)	1.25 (1.14, 1.37)**	1.16 (1.09, 1.23)**
Overlap	2.2 (2, 2.3)	1.43 (1.26, 1.61)**	1.2 (1.11, 1.29)**
Age (Ref. 15–19)	1	1	1
20–24	1.54 (1.46, 1.63)	1.96 (1.73, 2.2)**	1.85 (1.66, 2.1)**
25–29	2.7 (2.6, 2.9)	4.4 (3.9, 5)**	3.8 (3.4, 4.2)**
30–34	4.2 (4, 4.4)	7.3 (6.5, 8.3)**	5.9 (5.3, 6.5)**
35–39	5.8 (5.6, 6.2)	11.3 (9.9, 12.7)**	8.6 (7.7, 9.6)**
40–44	7.9 (7.5, 6.2)	15.9 (14, 18)**	11.6 (10.4, 12.8)**
45–49	10.2 (9.6, 10.7)	20.8 (18.4, 23.7)**	14.5 (13.1, 16.1)**
Education (Ref. No education)	1	1	1
Primary	0.66 (0.65, 0.67)	0.82 (0.79, 0.86)**	0.85 (0.83, 0.87)**
Secondary	0.33 (0.32, 0.33)	0.6 (0.57, 0.64)**	0.64 (0.62, 0.67)**
Higher	0.19 (0.18, 0.2)	0.35 (0.31, 0.4)**	0.4 (0.36, 0.44)**
Household size (Ref. > 6)	1	1	1
4–6	1.19 (1.16, 1.22)	0.86 (0.81, 0.9)**	0.91 (0.88, 0.94)**
≤3	1.58 (1.55, 1.62)	0.84 (0.79, 0.88)**	0.86 (0.83, 0.89)**
Relationship to household head (Ref. Head)	1	1	1
Wife	1.08 (1.06, 1.1)	1.21 (1.17, 1.27)**	1.16 (1.13, 1.2)**
Other	0.51 (0.5, 0.52)	1.19 (1.11, 1.27)**	1.15 (1.1, 1.2)**
Toilet facility (Ref. Improved)	1	1	1
Unimproved	1.51 (1.84, 1.54)	1.14 (1.09, 1.19)**	1.09 (1.06, 1.12)**
Open diffusion	1.95 (1.91, 1.99)	1.08 (1.02, 1.14)**	1.03 (0.99, 1.07)
Wealth of household (Ref. poorest)	1	1	1
Poorer	0.91 (0.89, 0.93)	0.88 (0.83, 0.92)**	0.93 (0.9, 0.96)**
Middle	0.79 (0.78, 0.81)	0.84 (0.8, 0.89)**	0.88 (0.85, 0.91)**
Richer	0.65 (0.64, 0.67)	0.74 (0.7, 0.89)**	0.8 (0.77, 0.83)**
Richest	0.41 (0.4, 0.42)	0.61 (0.56, 0.66)**	0.66 (0.62, 0.69)**
Age at first birth (Ref. < 18)	1	1	1
18–20	0.64 (0.63, 0.66)	0.62 (0.6, 0.64)**	0.69 (0.67, 0.71)**
> 20	0.46 (0.45, 0.47)	0.36 (0.34, 0.37)**	0.46 (0.44, 0.47)**
Number of union (Once)	1	1	1
More than once	1.9 (1.87, 1.94)	1.31 (1.25, 1.36)**	1.17 (1.14, 1.2)**
**Higher (community) level characteristics**			
Health facility distance (Ref A big problem)	1	1	1
Not a big problem	0.81 (0.8, 0.82)	1.02 (0.98, 1.05)	1.01 (0.99, 1.04)
Residence (Ref. Urban)	1	1	1
Rural	1.75 (1.72, 1.76)	1.17 (1.1, 1.24)**	1.16 (1.1, 1.2)**
Source of drink water (Ref. Improved)	1	1	1
Unimproved	1.46 (1.43, 1.48)	1.07 (1.02, 1.12)**	1.07 (1.03, 1.1)**
Ecology (Ref. Highland/ > 2300)	1	1	1
Temperate (1501–2300 masl)	0.83 (0.76, 0.89)	1.18 (1.01, 1.38)**	1.16 (1.04, 1.31)**
Lowland (501–1500 masl)	0.91 (0.84, 0.98)	1.35 (1.16, 1.57)**	1.34 (1.2, 1.5)**
Subtropical (< 501 masl)	0.97 (0.9, 1.04)	1.42 (1.22, 1.65)**	1.39 (1.24, 1.55)**

^a^AOR: adjusted odds ratio for mixed effect logistic regression.

^b^AIRR: adjusted incidence rate ratio for mixed effect negative binomial regression.

## Materials and methods

### Study area

The study focuses on the recent use of evidence in the Demographic and Health Surveys (DHS) and Multiple Indicator Cluster Surveys (MICS) from 42 SSA countries.

### Data source

This data analysed from SSA countries analysed using surveys conducted on the recent DHS/MICS data of each country. Surveys include the total number of childbirths and deaths of each woman. Most countries Global Positioning System (GPS) data included in the dataset.

### Dependent variables

The outcomes of interest are women who suffer from child death and the number of child deaths.

#### Inclusion/exclusion criteria

Women at fertile age (15–49) included in the study, whereas women who have not give birth exclude from the study and the child included in this study were from those women who fulfilled this criteria.

### Data processing and analysis

#### Hot spot analysis

Based on 95% confidence interval (CI), hot spot is defined as clustering of high value of child death and number of mothers who lost their child with a Z score ≥ 2 and a P value < 0.05; Cold spot is a cluster of fewer child deaths and fewer mothers who have lost their child due to death with a Z score ≤ − 2 and a P value < 0.05; and a Z score close to zero means no spatial clustering. Mapping hot spot analysis is applied to local G* statistics and is used to identify and display clusters of high prevalence (hot spot) regions and low prevalence (cold spot) regions^30^.

#### Spatial scan statistical analysis

Kulldoruff's Scan Statistic was used to analyse the spatial distribution of the prevalence of child death and mothers who had lost their child. A purely spatial scan statistic used to identify areas with higher number of child deaths and mothers lost their child. Spatially significant higher and lower aggregate concentrations were identified and circular windows were observed^31–34^. With the discreet Poisson model, the number of cases in each cluster (enumeration area) has been estimated^30^.

#### Spatial interpolation

Spatial interpolation using ordinary kriging has been used to predict the prevalence of non-mediated areas from measured areas^35^.

### Factor analysis

Due to the nature of the data, the risk factor of child death and mothers who suffer child death were only maternal/household and community characteristics. Because in both cases, the starting point of the problem was child death. Improved maternal health at the same time reduces the number of child deaths and mothers suffering from child deaths^36^.

#### Mixed effect model

Multi-level regression was conducted to assess factors related to maternal and community-level characteristics of child death and mothers suffering from child death. Multilevel analysis was considered appropriate due to the hierarchical nature of the DHS data as well as the estimation of individual and community level effects^37^. Data organized in two ways, the first to analyse the burden of child death on mothers, the outcome variable is child loss or not, which has binary response and run logistic regression analysis. The second outcome is the number of child deaths. The response is a count (0, 1, 2, 3, …) that fits Poisson's regression. Mixed effect multi-level Poisson and logistic regression model use and consisted of two levels (individual (women) and community levels). The appropriateness of the mixed model is checked using Inter-Class Correlation. The Poisson regression has been checked over/under dispersion.
